# An important role for A20-binding inhibitor of nuclear factor-kB-1 (ABIN1) in inflammation-mediated endothelial dysfunction: an *in vivo* study in ABIN1 (D485N) mice

**DOI:** 10.1186/s13075-015-0543-3

**Published:** 2015-02-04

**Authors:** Naveed Akbar, Sambit Nanda, Jill Belch, Philip Cohen, Faisel Khan

**Affiliations:** Vascular and Inflammatory Diseases Research Unit, Medical Research Institute, Division of Cardiovascular and Diabetes Medicine, Ninewells Hospital and Medical School, University of Dundee, Dundee, DD1 9SY UK; MRC Protein Phosphorylation and Ubiquitylation Unit, Sir James Black Centre, College of Life Sciences, University of Dundee, Dundee, UK

## Abstract

**Introduction:**

The link between cardiovascular disease (CVD) and patients with chronic inflammation is not clearly understood. We examined a knock-in mouse expressing a poly-ubiquitin-binding-defective mutant of the protein ABIN1 (ABIN1(D485N)), which develops a systemic lupus erythematosus-like autoimmune disease because of the hyperactivation of IκB kinases (IκKs) and mitogen-activated protein kinases (MAPKs). These mice were used to determine the potential role of these signaling pathways in inflammation-mediated CVD development.

**Methods:**

Laser Doppler imaging in combination with the iontophoresis of vasoactive chemicals were used to assess endothelium-dependent vasodilatation *in vivo* in ABIN1 (D485N)) mutant defective (*n* = 29) and wild-type (WT) control (*n* = 26) mice. Measurements were made at baseline, and animals were subdivided to receive either chow or a proatherogenic diet for 4 weeks, after which, follow-up assessments were made. Paired and unpaired *t* tests, and ANOVA with *post hoc* Bonferroni correction were used for statistical significance at *P* <0.05.

**Results:**

Endothelium-dependent vasodilatation to acetylcholine was attenuated at 4 weeks in ABIN1(D485N)-chow-fed mice compared with age-matched WT-chow-fed mice (*P* <0.05). The magnitude of attenuation was similar to that observed in WT-cholesterol-fed animals (versus WT-chow, *P* <0.01). ABIN1(D485N)-cholesterol-fed mice had the poorest endothelium-dependent responses compared with other groups (*P* <0.001). ABIN1(D485N)-chow-fed mice had increased plasma interleukin-6 (IL-6) levels (versus WT-chow, *P* <0.001), and this was further elevated in ABIN1(D485N)-cholesterol-fed mice (versus ABIN1(D485N)-chow; *P* <0.05). IL-1α was significantly greater in all groups compared with WT-chow (*P* <0.01). ABIN1(D485N) mice showed significant cardiac hypertrophy (*P* <0.05).

**Conclusions:**

The ABIN(D485N) mice display endothelial dysfunction and cardiac hypertrophy, which is possibly mediated through IL-6 and, to a lesser degree, IL-1α. These results suggest that the ABIN1-mediated hyperactivation of IKKs and MAPKs might mediate chronic inflammation and CVD development.

## Introduction

The role of inflammation in cardiovascular disease (CVD) is evident in patients with chronic inflammatory conditions such as systemic lupus erythematosus (SLE) [1] and rheumatoid arthritis (RA) [2], in which the standardized mortality rate is higher [3] and probably due to accelerated atherosclerosis [4]. Patients who have RA or SLE show overexpression for numerous proinflammatory cytokines, including tumor necrosis factor (TNF) and C-reactive protein (CRP). Additionally, patients show significant leukocyte infiltration into the subendothelial space and have elevated levels of oxidative stress, which are key mediators of vascular dysfunction [5,6].

Significant disease associations have been shown between vascular function, high sensitive-CRP (hs-CRP), and interleukin-6 (IL-6) in RA and SLE patients [7,8], suggesting a link between systemic cytokine overexpression and endothelial dysfunction, an early event in the development of CVD. Because the significant elevation in CVD risk in RA and SLE patients is not fully explained by traditional risk factors, such as age, plasma cholesterol, and tobacco smoking [9], we need to better understand the mechanisms and underlying pathways that link chronic inflammatory diseases with increased CVD risk.

Numerous factors drive inflammation, including nuclear factor kappa-B (NF-κB). This transcription is activated by inflammatory stimuli and mediates the release of inflammatory molecules associated with atherosclerosis, including IL-6, IL-12, and TNF [10,11]. Activators of NF-κB include Toll-like receptors (TLRs), endothelin-1, reactive oxygen species (ROS), and oxidized lipids [12,13], which, through complex inflammatory pathways, induce gene expression and are proatherogenic.

Under basal conditions, NF-κB is maintained in the cytoplasm in an inactive state through inhibitors of κB (IκB). On activation, IκB rapidly undergoes phosphorylation and degradation, inducing nuclear translocation and gene expression. The A20-binding inhibitors of NF-κB (ABINs1-3) are suppressors of inflammation. Recent work suggests that ABIN1 restricts the activation of the canonic IKK complex and mitogen-activated protein kinases (MAPKs) by binding to Lys63-linked and linear ubiquitin chains [14].

Human polymorphisms in the gene encoding the ABIN1 protein have been identified and are associated with a predisposition for autoimmune disease [15]. The clinical manifestations of polymorphisms in ABIN1 are widespread and can resemble SLE [16,17]. Mutations in ABIN1 are associated with psoriasis, psoriatic arthritis, and importantly, in the context of CVD, He *et al*. [18] reported an enhanced risk of vasculitis. The TNIP1 (TNFAIP3-interacting protein 1) gene locus that encodes for the protein ABIN1 is associated with the predisposition to SLE [19] is also thought to be significantly associated with other inflammatory-mediated pathologies, including coronary heart disease and myocardial infarction [20-22]. Wolfrum *et al*. [23] studied mice that were haploinsufficient for A20, a protein that interacts with ABIN1, and backcrossed them onto the CVD-prone apolipoprotein E knockout mouse. These mice showed significant exacerbation in atherosclerotic lesion presentation mediated through NF-κB activation [23].

The important roles of ABINs in physiology are highlighted in gene-targeted knockout mice that display organ failure and premature death [24]. We previously reported on a knockin mouse in which wild-type ABIN1 was replaced by the polyubiquitin-binding-defective mutant ABIN1[D485N] [14]. Several types of immune cells from these mice show enhanced NF-κB and MAPK activation after TLR stimulation and display a SLE-like phenotype [14]. The ABIN1[D485N] knockin mice show significant expansion of myeloid cells in various organs [14]. Caster *et al*. [15] reported similarity between the ABIN1[D485N] mice and SLE patients in the context of lupus nephritis, a leading cause of morbidity and mortality in chronic inflammation. The onset of CVD relevant to this mutation has not been addressed.

We sought to establish the early cardiovascular consequences (before development of overt atherosclerosis and plaque formation) of an aberration in the homeostatic control of NF-κB and MAPK by studying the ABIN1[D485N] mutant. We assessed endothelial function as an early marker of CVD *in vivo*, the better to understand the link between modulators of inflammation and early development of CVD.

## Methods

### Mice

The ABIN1[D485N] mice were originally described on a 129SvJxC57B/6 background [14]. Animals were subsequently backcrossed on a C57B/6 background for at least eight generations. Mutant defective ABIN1[D485N] and wild-type (WT) mice used in these studies shared a common genetic background.

Prior approval was obtained from the institutional ethical review committee (University of Dundee Ethical Review Committee), and experimental interventions were carried out by UK Home Office personal license-holders under the authority of a Home Office project license. All mice were male. The ABIN1[D485N] mice were age matched to littermate WT controls. Animals were transferred from a barrier breeding facility to the experimental facility (where the necessary equipment was installed), at least 1 week before vascular-function testing, to allow acclimation and to avoid stress.

Group allocations were randomly assigned as follows: WT control mice fed normal rodent chow (SDS R&M No.1) (*n* = 15), WT mice on a specifically tailored proatherogenic diet (*n* = 14) (TD.01383 diet, Harlan-Teklad), ABIN1[D485N] mice on rodent chow (*n* = 12), and a proatherogenic diet (*n* = 14). Researchers were blinded to genotypes in the group allocations throughout the study.

### Vascular responses

Skin microvascular responses were measured *in vivo* at baseline (week 0) and 4 weeks later by using laser Doppler imaging (LDI) and iontophoresis of vasoactive chemicals, as described previously [25]. In brief, iontophoresis chambers were attached to the flanks of anesthetized mice (isoflurane in medical oxygen 1.5% to 2% delivered via a nose cone) by using double-sided adhesive tape.

### Endothelium-dependent responses

Baseline perfusion was normalized by preconstriction by using iontophoresis of a 1% solution of phenylephrine (PE) (Sigma-Aldrich). After this, a 2% solution of the endothelium-dependent vasodilator acetylcholine (ACh) (Sigma-Aldrich) was iontophoresed for 10 minutes, and the maximum vasodilatation measured by LDI. Perfusion was expressed in arbitrary perfusion units ± standard error (SEM) and calculated by using propriety software (Moor LDI software, version 5.2) as a percentage change over baseline.

### Maximum vasodilator response to localized skin heating

A skin-heating probe (VPH3; Moor Instruments) with a total surface area of 3.2 cm^2^ was used to assess maximal dilator capacity. Baseline measurements of skin perfusion were taken for 5 minutes, followed by localized heating of the skin to 44°C.

### Endothelium-independent responses

Endothelium-independent vasodilatation was assessed at 4 weeks only by using sodium nitroprusside (SNP) (Sigma-Aldrich) by following a similar protocol to that for ACh. SNP was iontophoresed for 10 minutes.

### Plasma cholesterol analysis

Plasma was used to quantify high-density lipoprotein (HDL), low-density, and very low-density lipoproteins (LDL/vLDL) fractions at week 4, by using a color metric assay (Abcam, Product code: ab655390), as detailed in the manufacturer’s instructions. Results are expressed as mg/dl ± SEM.

### Cytokine analysis

Plasma was analyzed by using custom Bio-Plex® Pro kits™ from BIO-RAD Laboratories at baseline and at 4 weeks for IL-1α, IL-6, and IL-10, as detailed in the manufacturer’s instructions. Results are expressed at pg/ml ± SEM.

### Cardiac hypertrophy

In cholesterol-fed animals only cardiac hypertrophy was determined by calculating the ratio: total heart weight (mg)/average length of tibia (mm).

### Statistical analysis

Data are expressed as group means ± SEM. Within- and between-group differences were compared by using paired, unpaired *t* tests and one-way ANOVA with *post hoc* Bonferroni correction when significant differences were found. Associations between microvascular responses, plasma measures of cytokines, plasma cholesterol (LDL/vLDL and HDL), spleen mass, and cardiac hypertrophy were tested by using Pearson correlation coefficients in the software package PASW Statistic (Version 21). The null hypothesis was rejected at *P* <0.05.

## Results

### Body weight

**Figure 1:**
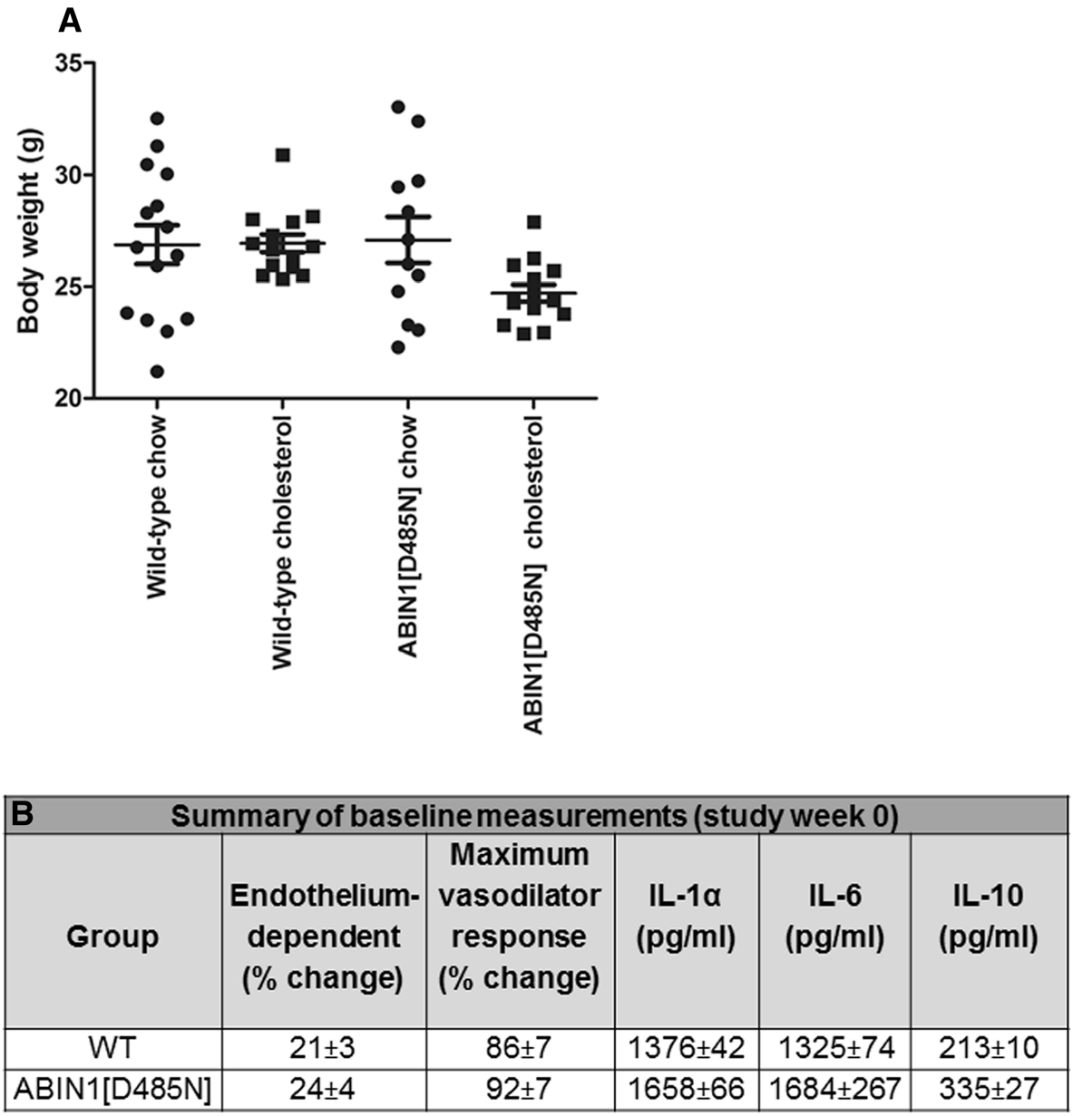
**Body weight. (A)** Body weights (study week 4) in WT-chow (*n* = 15), WT-cholesterol (*n* = 14), ABIN1[D485N]-chow (*n* = 12) and ABIN1[D485N]-cholesterol (*n* = 14) fed mice. One-way ANOVA with *post hoc* Bonferroni correction. Grams (g) ± SE. **B**: Summary of baseline measurements (study week 0). Baseline (12 weeks of age) plasma cytokines for IL-1α, IL-10, and IL-6 in WT (*n* = 10) and ABIN1[D485N] (*n* = 9) mice (pg/ml ± SEM). Microvascular responses in WT and ABIN1[D485N] animals: endothelium-dependent responses (WT *n* = 14, ABIN1[D485N] *n* = 10), and maximal dilator capacity (WT *n* = 17, ABIN1[D485N] *n* = 15) (% change ± SEM).

Body weight increased in all groups over the study period, and after 4 weeks’ feeding, no significant differences were found among the different mouse groups (Figure 1).

Study baseline data for plasma cytokines and vascular responses are summarized in Figure 1B.

### Baseline cytokines

**Figure 2:**
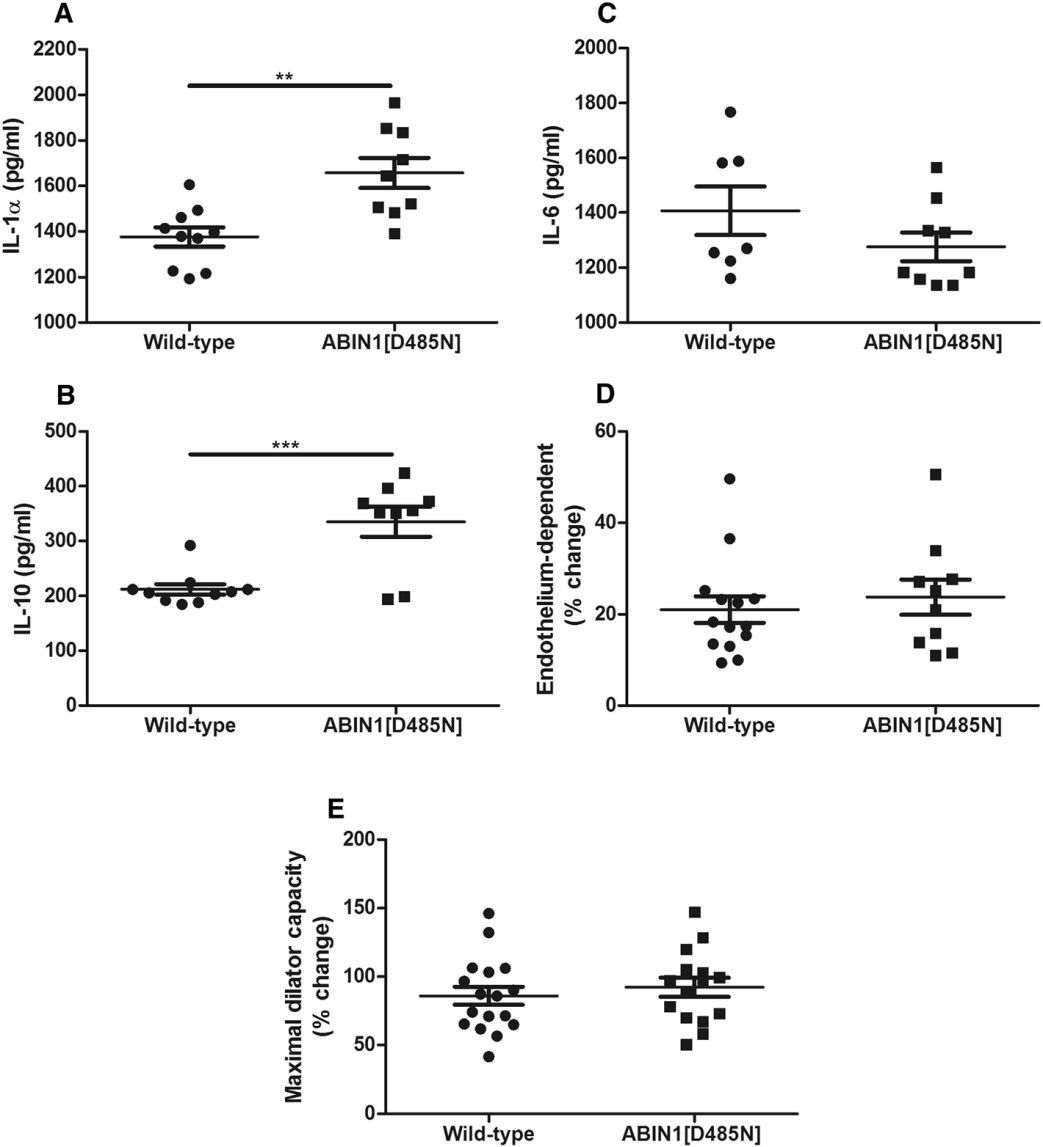
**Study baseline measurements.** Baseline (12 weeks of age) plasma cytokines for **(A)** IL-1α **(B)** IL-10, and **(C)** IL-6 in WT (*n* = 10) and ABIN1[D485N] (*n* = 9) mice (pg/ml ± SEM). Microvascular responses in WT and ABIN1[D485N] animals: **(D)** endothelium-dependent responses (WT *n* = 14, ABIN1[D485N] *n* = 10), and **(E)** maximal dilator capacity (WT *n* = 17, ABIN1[D485N] *n* = 15) (% change ± SEM). Unpaired Student *t* test. **P < 0.01, ***P < 0.001.

Significant differences for baseline inflammatory markers were found between WT and ABIN1[D485N] mice. IL-1α was significantly greater in ABIN1[D485N] mice (WT 1,376 ± 42 pg/ml versus ABIN1[D485N] 1,658 ± 66 pg/ml; *P* <0.01), as was antiinflammatory IL-10 (WT 213 ± 10 pg/ml versus ABIN1[D485N] 335 ± 27 pg/ml; *P* <0.001) (Figure 2A, B respectively). Levels of IL-6 were not significantly different between the groups (WT 1,325 ± 74 pg/ml versus ABIN1[D485N] 1,684 ± 267 pg/ml) (Figure 2C).

### Baseline vascular responses

Baseline vascular responses were not significantly different between WT and ABIN1[D485N] mice for endothelium-dependent responses or maximal dilator capacity (ACh: WT 21% ± 3% change ABIN1[D485N] 24% ± 4%; maximal dilator capacity: WT 86% ± 7% ABIN1[D485N] 92% ± 7%) (Figure 2D/E, respectively).

### Plasma cholesterol

**Figure 3:**
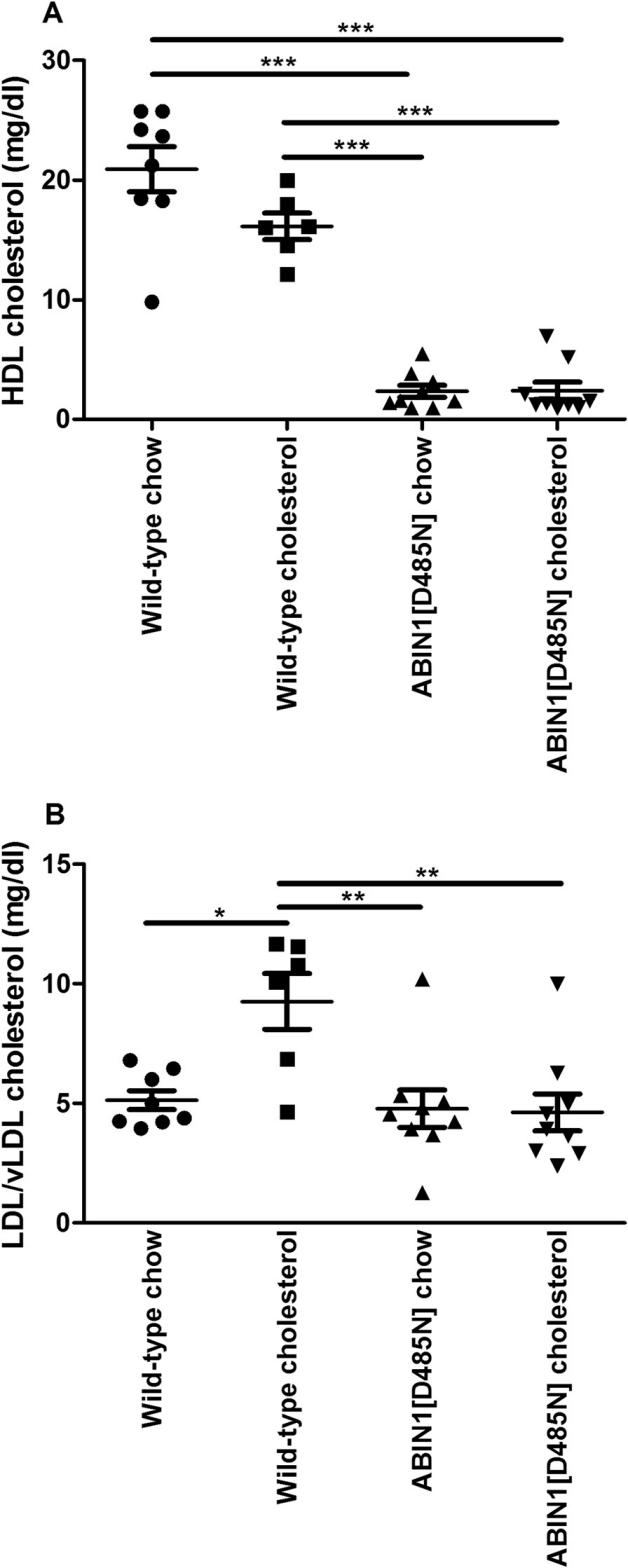
**Plasma cholesterol.** Week 4 plasma levels of **(A)** high-density lipoproteins (HDLs) in WT-chow (*n* = 8), WT-cholesterol (*n* = 6), ABIN1[D485N]-chow (*n* = 9), and ABIN1[D485N]-cholesterol-fed mice (*n* = 9); and **(B)** low-density lipoproteins and very low-density lipoproteins (LDL/vLDL) in the same animals (mg/dl ± SEM). One-way ANOVA with *post hoc* Bonferroni correction. *P <0.05, **P <0.01. ***P <0.001.

Measurements of HDL cholesterol at 4 weeks showed that WT-chow (21 ± 2 mg/dl)-fed mice had significantly greater HDL levels compared with ABIN1[D485N]-chow (2.0 ± 0.5 mg/dl, *P* <0.001) and ABIN1[D485N]-cholesterol (2.0 ± 0.7 mg/dl, *P* <0.001)-fed mice (Figure 3A). HDL levels were significantly greater in WT-cholesterol mice when compared with ABIN1[D485N]-chow (*P* <0.001) and ABIN1[D485N]-cholesterol (*P* <0.001) mice (Figure 3A).

WT-cholesterol-fed mice had significantly greater LDL/vLDL compared with the other groups (versus WT-chow, *P* < 0.05, versus ABIN1[D485N]-chow *P* <0.01, versus ABIN1(D485N)-cholesterol *P* < 0.01) at 4 weeks (Figure 3B).

### Cytokines at 4-week follow-up

**Figure 4:**
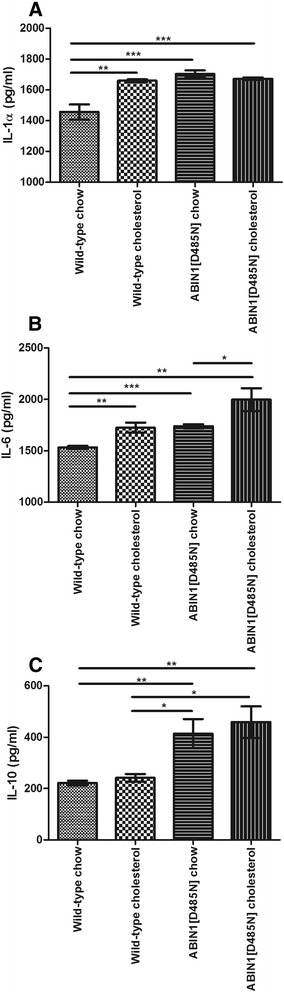
**Follow-up plasma cytokines.** Plasma inflammatory markers at 4 weeks in WT-chow (*n* = 10), WT-cholesterol (*n* = 7), ABIN1[D485N]-chow (*n* = 10), and ABIN1[D485N]-cholesterol (*n* = 10)-fed animals for **(A)** IL-1α, **(B)** IL-6, and **(C)** IL-10 (pg/ml ± SEM). One-way ANOVA with *post hoc* Bonferroni correction *P <0.05, **P <0.01, ***P <0.001.

Levels of IL-1α (1,457 ± 49 pg/ml), IL-6 (1,393 ± 53 pg/ml), and IL-10 (222 ± 9 pg/ml) did not change significantly in WT-chow-fed mice over time compared with baseline values. Cholesterol feeding in WT mice significantly increased IL-1α (1,659 ± 10 pg/ml) and IL-6 (1,726 ± 4 pg/ml), compared with baseline values (*P* <0.001, *P* <0.001, respectively). IL-6 and IL-1α were significantly greater in WT-cholesterol-fed mice compared with age-matched WT-chow-fed mice (Figure 4A, B, respectively). No significant differences in IL-10 were found between cholesterol-fed WT mice (230 ± 11 pg/ml) and age-matched WT-chow (Figure 4C). Levels of IL-1α did not change significantly in ABIN1[D485N]-chow-fed mice (1,704 ± 22 pg/ml), but remained significantly greater than age-matched WT-chow-fed mice (*P* <0.001). Cholesterol feeding in ABIN1[D485N] mice did not result in a further significant change in IL-1α (1,672 ± 8 pg/ml) (Figure 4A), but levels remained significantly greater than in age-matched WT-chow mice (*P* <0.001).

ABIN1[D485N]-chow-fed animals displayed a significant increase in IL-6 compared with baseline values (1,739 ± 21 pg/ml, *P* <0.001). IL-6 at 4 weeks was significantly greater in ABIN1[D485N]-chow and ABIN1[D485N]-cholesterol-fed mice compared with WT-chow (Figure 4B). ABIN1[D485N]-cholesterol-fed mice had greater IL-6 levels compared with ABIN1[D485N]-chow (*P* <0.05) (Figure 4B).

Plasma measurements of IL-10 at 4 weeks did not significantly change in ABIN1[D485N] mice compared with baseline values. IL-10 levels remained significantly greater in ABIN1[D485N] mice compared with WT-chow and WT-cholesterol-fed mice (Figure 4C).

### Vascular responses at 4 weeks

#### Endothelium-dependent responses

WT animals on normal rodent-chow diet did not show significant changes in ACh-mediated vasodilatation over the study duration (baseline, 22% ± 4% change versus 4 weeks 22% ± 4% change).

**Figure 5:**
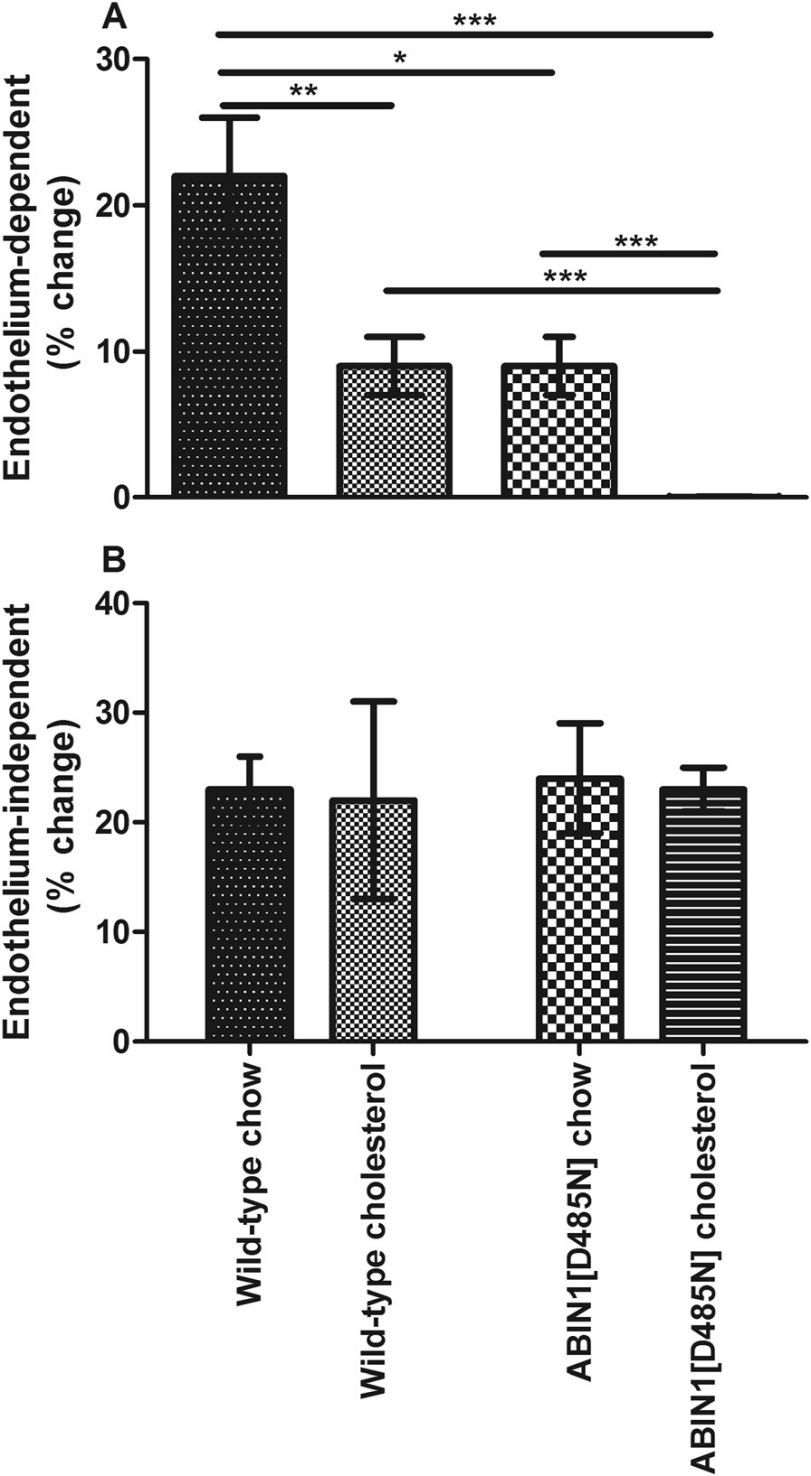
**Follow-up vascular function.** Microvascular responses at 4 weeks to **(A)** endothelium-dependent acetylcholine in WT-chow (*n* = 11), WT-cholesterol (*n* = 11), ABIN1[D485N]-chow (*n* = 10), and ABIN1[D485N]-cholesterol (*n* = 10)-fed mice; and **(B)** endothelium-independent sodium nitroprusside in the same mice (% change ± SEM). One-way ANOVA with *post hoc* Bonferroni correction. *P <0.05; **P <0.01; ***P <0.001.

WT mice fed a proatherogenic diet for 4 weeks showed a decrease in ACh-mediated vasodilatation compared with baseline values (baseline, 20% ± 3% change versus 4 weeks 9% ± 2% change; *P* <0.01) and WT age-matched mice on rodent chow (*P* <0.001). ACh-mediated vasodilatation in ABIN1[D485N]-chow (9% ± 2% change)-fed mice were significantly attenuated compared with values at baseline (*P* <0.001) and were significantly lower than age-matched WT mice on a chow diet (*P* <0.05) at 4 weeks, but were similar in magnitude to those observed in WT-cholesterol-fed mice (Figure 5A). Cholesterol feeding in ABIN1[D485N] mice further attenuated ACh-mediated vasodilatation (0.03% ± 0.03% change) compared with age-matched ABIN1[D485N]-chow-fed mice (*P* <0.001). ACh-mediated vasodilatation was significantly lower in ABIN1[D485N]-cholesterol-fed animals compared with WT-chow (*P* <0.001) and WT-cholesterol (*P* <0.001)-fed mice (Figure 5A).

#### Endothelium-independent responses

At 4 weeks, endothelium-independent responses were not significantly different among the groups (Figure 5B).

### Cardiac hypertrophy

**Figure 6:**
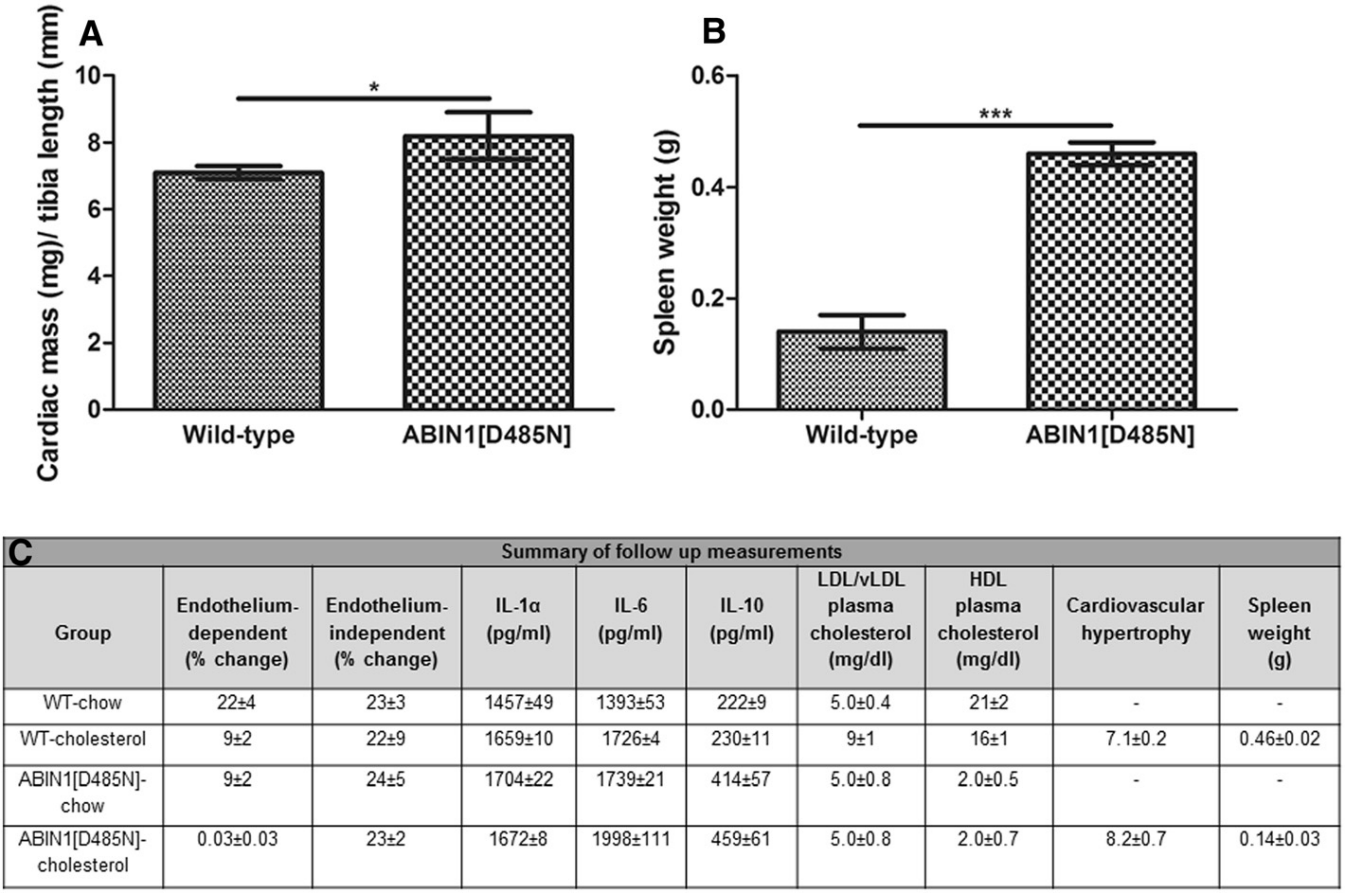
**Cardiac and spleen mass. (A)** Cardiac hypertrophy measurements in WT (*n* = 14) and ABIN1[D485N] mice (*n* = 13) cholesterol-fed mice: cardiac mass (mg) versus tibia length (mm) ± SEM **(A)**. **(B)** Spleen mass (g ± SEM) in WT (*n* = 6) and ABIN1[D485N] (*n* = 7) cholesterol-fed animals. Unpaired Student *t* test. *P <0.05, ***P <0.001. **(** **C)** Summary of follow-up measurements: High-density lipoproteins (HDLs) in WT-chow (*n* = 8), WT-cholesterol (*n* = 6), ABIN1[D485N]-chow (*n* = 9), and ABIN1[D485N]-cholesterol- fed mice (*n* = 9) and low-density lipoproteins and very-low-density lipoproteins (LDL/vLDL) in the same animals (mg/dl ± SEM). Plasma inflammatory markers at 4 weeks in WT-chow (*n* = 10), WT-cholesterol (*n* = 7), ABIN1[D485N]-chow (*n* = 10), and ABIN1[D485N]-cholesterol (*n* = 10)-fed animals for **(A)** IL-1α, **(B)** IL-6, and **(C)** IL-10 (pg/ml ± SEM). Microvascular responses at 4 weeks to endothelium-dependent acetylcholine in WT-chow (*n* = 11), WT-cholesterol (*n* = 11), ABIN1[D485N]-chow (*n* = 10) and ABIN1[D485N]-cholesterol (n = 10) fed mice and endothelium-independent sodium nitroprusside in the same mice (% change ± SEM). Cardiac hypertrophy measurements in WT (*n* = 14) and ABIN1[D485N] mice (*n* = 13) cholesterol-fed mice and spleen mass (gram ± SEM) in WT (*n* = 6) and ABIN1[D485N] (*n* = 7) cholesterol-fed animals. Results are expressed as group means ± SEM.

Significant differences for cardiac hypertrophy were found in ABIN1[D485N] mice (WT 7.1 ± 0.2 versus ABIN1[D485N] 8.2 ± 0.7; *P* <0.05) (Figure 6A).

### Spleen weight

Spleen mass was greater in cholesterol-fed ABIN1[D485N] mice (0.46 ± 0.02 g) compared with WT-cholesterol-fed mice (0.14 ± 0.03 g) (*P* <0.001) (Figure 6B).

Follow-up assessment of vascular responses and plasma cytokines, plasma cholesterol, measurements of cardiac hypertrophy, and spleen weight are summarized in Figure 6C.

### Correlations

No significant correlations were noted between baseline vascular responses and plasma cytokines. Conversely, significant associations were found at 4 weeks. ACh-mediated vasodilatation at 4 weeks correlated negatively with spleen mass (r = −0.722; *P* <0.01) and with IL-1α (r = −0.0764, *P* <0.01). HDL correlated negatively with IL-1α (r = −0.501, *P* <0.01) and IL-6 (r = −0.558, *P* <0.001).

## Discussion

Here we describe for the first time the onset of early CVD (endothelial dysfunction) in the polyubiquitin-binding-defective ABIN1[D485N] mice, a phenotype that is further exacerbated by cholesterol feeding. We found no significant differences in endothelium-independent responses, suggesting that vascular smooth muscle activity was not compromised and indicates localized damage to the endothelium. We further report a significant reduction in plasma HDL of ABIN1[D485N] mice, an established risk factor for CVD.

We previously reported that ABIN1[D485N] mice have enhanced IKK and MAPK activity in B cells, bone marrow-derived macrophages, and dendritic cells and display significant expansion of myeloid cells in spleen and lymph nodes [14]. Consequently, these mice bear an SLE-like phenotype as early as 3 to 4 months of age. Endothelial dysfunction is an early event in the development of CVD, and we show in this study that it is present in ABIN1[D485N] mice at 4 months of age. Endothelial dysfunction was further exacerbated by dietary cholesterol, showing elevated dysfunction when risk factors are combined (chronic inflammation and cholesterol).

Selective inhibition of NF-κB from endothelial cells has been shown to be protective against atherosclerotic lesion formation in CVD-prone apolipoprotein E knockout mice [26]. Our data support the notion that increased IKK activity, and hence increased NF-ĸB activation, has significant negative effects on the cardiovascular system. The activation of TLRs, in particular, TLR-2 and TLR-4, is associated with atherogenesis, whereas blockade of this signaling, achieved by amelioration of the myeloid differentiation primary-response gene 88 (MyD88), has shown atheroprotection [27]. Similarly, the SLE phenotype of the ABIN1[D485N] mice is abrogated when they are expressed on an MyD88-deficient background [14], indicative of overlap in the signaling pathways involved in the development of SLE and CVD.

We found a significant increase in IL-1α in plasma of ABIN1[D485N] mice at the study baseline, although no apparent difference occurred in vascular responses between the two groups, suggesting that the relative differences and duration of change was not sufficient to affect vascular function at this time point. The differences in inflammatory markers between ABIN1[D485N] and WT mice are presumably mediated by the hyperactivation of NF-ĸB and MAPKs. Commensal gut flora can activate TLRs and stimulate NF-ĸB [28,29], predisposing to chronic inflammation in ABIN1[D485N] mice.

The exact mechanism responsible for the onset of endothelial dysfunction in ABIN1[D485N] mice requires further investigation; however, this may be mediated by IL-6. We found a significant increase in IL-6 over time in ABIN1[D485N] mice, and this was further exacerbated by cholesterol feeding in ABIN1[D485N] mice, which displayed the poorest endothelium-dependent responses. IL-6 is an established cardiovascular risk factor [30,31]. Levels of IL-6 in WT-cholesterol mice at the end point were similar to those observed in ABIN1[D485N]-chow mice, even though the latter group was not exposed to a major cardiovascular risk factor (cholesterol). A negative association between vascular responses and plasma levels of IL-6 was described previously [32]. Taken together, these data suggest that the mutation in ABIN1[D485N] mice predisposes to CVD in part via increasing levels of IL-6.

IL-6 can inhibit activation of endothelial nitric oxide synthase (eNOS) and attenuate vasodilation by increasing the half-life of caveolin-1, resulting in more eNOS binding and reducing the bioavailability of NO [33]. NO is produced basally in the vascular system and maintains vascular tone. A loss in the bioavailability of NO is associated with diseases such as hypertension [34] and is regarded as an early phase of atherosclerotic plaque formation [35]. Importantly, endothelial dysfunction mediated through NO loss is an early hallmark event in atherosclerotic plaque formation, preceding vascular damage. The stimulated endothelium can express a number of vascular cell-adhesion molecules, and this in turn facilitates the movement of leukocytes into the arterial intima, a prerequisite for atherosclerotic plaque formation.

We previously reported that skin microvascular responses to ACh are mediated through the bioavailability NO and that this is diminished by cholesterol feeding in WT mice [25]. The loss of NO in the peripheral skin microcirculation increases total peripheral resistance. Reduced lumen diameter through attenuated vasodilatation (diminished NO bioavailability) can lead to development of left ventricular hypertrophy, an adaptive response to increased cardiac load (greater force is needed to pulsate blood through narrow arteries). This adaptation is essential for survival, and inhibition of cardiac hypertrophy in mice shows increased mortality through pressure overload and heart failure [36]. Thus, we conclude that cardiac hypertrophy in ABIN1[D485N]-cholesterol-fed mice may be an adaptive response to diminished peripheral microvascular function.

It is important to note that under pathophysiologic conditions, cytokines are released from numerous cell types, including activated endothelial cells. Stimulated endothelial cells express IL-1α. IL-1α is associated with CVD [37] and can contribute to atherosclerosis through the expression of cell-adhesion molecules (vascular cell-adhesion molecule-1 and intracellular adhesion molecule-1). Adhesion molecules are needed for the tethering of monocytes to the endothelial lining, for subsequent transmigration into the subendothelial space, an early phase in atherosclerotic plaque formation.

Surprisingly, plasma levels of the antiinflammatory IL-10 were significantly greater in ABIN1[D485N] mice, an unreported finding. The exact mechanism and significance of these remains unknown. IL-10 can be synthesised by macrophages and attenuates proinflammatory cytokine expression through a JAK/STAT3 pathway [38]. Forsberg *et al.* [39] previously reported similar findings, showing increased levels of IL-10 in intraepithelial lymphocytes in the context of celiac disease. The ability to maintain these elevated levels of IL-10 is of particular interest, and it must be established whether they can be sustained over a longer period (with greater age), and to establish whether ablation or sequestering of endogenous IL-10 in ABIN1[DN485] mice would further exacerbate the observed pathology in this and previous studies.

IL-10 levels are antiatherogenic, facilitating the uptake and efflux of cholesterol, which in turn is associated with reduced cell death and regression of atherosclerotic lesions [40]. This may in part explain why ABIN1[D485N]-cholesterol-fed mice, despite being fed dietary cholesterol for 4 weeks, did not display elevated LDL/vLDL, unlike WT-cholesterol-fed mice. Pinderski *et al*. [41] previously reported a lower plasma cholesterol level in animals overexpressing IL-10 compared with both C57/B6 WT mice and homozygous IL-10-null animals fed cholesterol, although these observations were not statistically significant. In healthy individuals, the efflux of cholesterol from the arterial intima is modulated by HDL and prevents lipid oxidation. Thus, despite profound reductions in HDL ABIN1[D485N] mice may be able to efflux LDL/vLDL cholesterol through an IL-10-dependent mechanism to prevent significant accumulation in the bloodstream. It remains unknown why the ABIN1[D485N] mutant defective mice show depleted levels of HDL in their plasma at 16 weeks of age. We must establish whether cholesterol metabolism in these ABIN1[D485N] mice is affected primarily because of the mutant gene, or whether the progressive development of the SLE-like phenotype in ABIN1[D485N] mice successively affects plasma HDL levels significantly.

We aimed to establish a role for a mutation in ABIN1 in inflammatory-induced CVD development through assessment of endothelial function and measurement of systemic cytokine expression. Understanding the pathophysiological mechanisms and pathways responsible for early development of CVD in ABIN1[D485N] mice has potentially important clinical implications. The induction of chronic inflammation, endothelial dysfunction, and cardiac hypertrophy by a single protein malformation highlights the need for selective therapeutic targets of inflammation to limit multiorgan disease, and the ABIN1 pathway might be one potential therapeutic target. It is important that, by using a similar experimental approach in humans, we previously showed that RA patients have attenuated endothelium-dependent responses in the skin microcirculation, and that the degree of attenuation is related to the expression of systemic inflammatory cytokines [8], findings that are similar to those in the present study. Because endothelium-dependent responses in the microcirculation of the skin are indicative of defective coronary function [42] and future cardiovascular sequelae before clinical presentation [43], the findings from the present study point to a potentially important role for ABIN1 in the development and progression of inflammation-induced CVD. The findings in this study are in agreement with irregularities in the NF-ĸB pathway that were previously implicated for the onset of CVD [18,20-22].

## Conclusions

In conclusion, we believe this to be the first *in vivo* observation to document the early development of CVD (endothelial dysfunction) as a result of a single protein mutation involved in NF-ĸB signaling, relevant to previously reported clinical genome-wide association studies for SLE. Our data suggest that ABIN1 dysfunction could be mechanistically involved in the early development of inflammation-induced CVD risk.

## Abbreviations

ABINs: A20-binding inhibitors of NF-κB
ACh: acetylcholine
CRP: C-reactive protein
CVD: cardiovascular disease
eNOS: endothelial nitric oxide synthase
HDL: high density lipoprotein
hs-CRP: high sensitive-CRP
IκB: inhibitor of KB
IKKs: inhibitors of κB kinases
IL-6: interleukin-6
LDI: laser Doppler imaging
LDL/vLDL: low-density and very-low-density lipoprotein
MAPK: mitogen-activated protein kinase
MyD88: myeloid differentiation primary response gene (88)
NF-κB: nuclear factor kappa B
PE: phenylephrine
RA: rheumatoid arthritis
ROS: reactive oxygen species
SEM: standard error of the mean
SLE: systemic lupus erythematosus
TLR: Toll-like receptor
TNF: tumor necrosis factor
TNIP1: TNFAIP3-interacting protein 1
WT: wild-type

## References

[1] Lopez-Pedrera C, Aguirre MA, Barbarroja N, Cuadrado MJ (2010). Accelerated atherosclerosis in systemic lupus erythematosus: role of proinflammatory cytokines and therapeutic approaches. J Biomed Biotechnol..

[2] Szekanecz Z, Kerekes G, Der H, Sandor H, Szabo Z, Vegvari A (2007). Accelerated atherosclerosis in rheumatoid arthritis. Ann N Y Acad Sci..

[3] Yurkovich M, Vostretsova K, Chen W, Avina-Zubieta JA (2013). Overall and cause-specific mortality in patients with systemic lupus erythematosus: a meta-analysis of observational studies. Arthritis Care..

[4] Shoenfeld Y, Gerli R, Doria A, Matsuura E, Cerinic MM, Ronda N (2005). Accelerated atherosclerosis in autoimmune rheumatic diseases. Circulation..

[5] Ku IA, Imboden JB, Hsue PY, Ganz P (2009). Rheumatoid arthritis: model of systemic inflammation driving atherosclerosis. Circulation..

[6] Full LE, Ruisanchez C, Monaco C (2009). The inextricable link between atherosclerosis and prototypical inflammatory diseases rheumatoid arthritis and systemic lupus erythematosus. Arthritis Res Ther..

[7] Barbulescu AL, Vreju F, Cojocaru-Gofita IR, Musetescu EA, Ciurea LP (2012). Impaired arterial stiffness in systemic lupus erythematosus: correlations with inflammation markers. Curr Health Sci J..

[8] Galarraga B, Khan F, Kumar P, Pullar T, Belch JJ (2008). C-reactive protein: the underlying cause of microvascular dysfunction in rheumatoid arthritis. Rheumatology (Oxford)..

[9] Esdaile JM, Abrahamowicz M, Grodzicky T, Li Y, Panaritis C, du Berger R (2001). Traditional Framingham risk factors fail to fully account for accelerated atherosclerosis in systemic lupus erythematosus. Arthritis Rheum..

[10] Huber SA, Sakkinen P, Conze D, Hardin N, Tracy N (1999). Interleukin-6 exacerbates early atherosclerosis in mice. Arterioscler Thromb Vas..

[11] Boesten LS, Zadelaar AS, van Nieuwkoop A, Gijbels JJM, de Winther PJM, Havekes ML (2005). Tumor necrosis factor-alpha promotes atherosclerotic lesion progression in APOE*3-Leiden transgenic mice. Cardiovasc Res..

[12] Browatzki M, Schmidt J, Kubler W, Kranzhofer R (2000). Endothelin-1 induces interleukin-6 release via activation of the transcription factor NF-kappaB in human vascular smooth muscle cells. Basic Res Cardiol..

[13] Cominacini L, Pasini AF, Garbin U, Davoli A, Tosetti ML, Campagnola M (2000). Oxidized low density lipoprotein (ox-LDL) binding to ox-LDL receptor-1 in endothelial cells induces the activation of NF-kappaB through an increased production of intracellular reactive oxygen species. J Bio Chem..

[14] Nanda SK, Venigalla RK, Ordureau A, Patterson-Kane JC, Powell DW, Toth R (2011). Polyubiquitin binding to ABIN1 is required to prevent autoimmunity. J Exp Med..

[15] Caster DJ, Korte EA, Nanda SK, McLeish KR, Oliver RK, Sheehan RM (2013). ABIN1 dysfunction as a genetic basis for lupus nephritis. J Am Soc Nephrol..

[16] Vaughn SE, Kottyan LC, Munroe ME, Harley JB (2012). Genetic susceptibility to lupus: the biological basis of genetic risk found in B cell signaling pathways. J Leukoc Biol..

[17] Adrianto I, Wang S, Wiley GB, Lessard CJ, Kelly JA, Adler AJ (2012). Association of two independent functional risk haplotypes in TNIP1 with systemic lupus erythematosus. Arthritis Rheum..

[18] He CF, Liu YS, Cheng YL, Gao GP, Pan TM, Han JW (2010). TNIP1, SLC15A4, ETS1, RasGRP3 and IKZF1 are associated with clinical features of systemic lupus erythematosus in a Chinese Han population. Lupus..

[19] Vereecke L, Beyaert R, van Loo G (2009). The ubiquitin-editing enzyme A20 (TNFAIP3) is a central regulator of immunopathology. Trends Immunol..

[20] Ozaki K, Ohnishi Y, Iida A, Sekine A, Yamada R, Tsunoda T (2002). Functional SNPs in the lymphotoxin-alpha gene that are associated with susceptibility to myocardial infarction. Nat Genet..

[21] Ozaki K, Tanaka T (2005). Genome-wide association study to identify SNPs conferring risk of myocardial infarction and their functional analyses. Cell Mol Life Sci..

[22] Boonyasrisawat W, Eberle D, Bacci S, Zhang YY, Nolan D, Gervino EV (2007). Tag polymorphisms at the A20 (TNFAIP3) locus are associated with lower gene expression and increased risk of coronary artery disease in type 2 diabetes. Diabetes..

[23] Wolfrum S, Teupser D, Tan M, Chen KY, Breslow JL (2007). The protective effect of A20 on atherosclerosis in apolipoprotein E-deficient mice is associated with reduced expression of NF-kappaB target genes. Proc Natl Acad Sci U S A..

[24] Zhou J, Wu R, High AA, Slaughter CA, Finkelstein D, Rehg JE (2011). A20-binding inhibitor of NF-kappaB (ABIN1) controls Toll-like receptor-mediated CCAAT/enhancer-binding protein beta activation and protects from inflammatory disease. Proc Natl Acad Sci U S A..

[25] Belch JJ, Akbar N, Alapati V, Alapati V, Petrie J, Arthur S (2013). Longitudinal assessment of endothelial function in the microvasculature of mice in-vivo. Microvasc Res..

[26] Gareus R, Kotsaki E, Xanthoulea S (2008). Va der Made I, Gijbels JJM, et al. Endothelial cell-specific NF-kappaB inhibition protects mice from atherosclerosis. Cell Metab..

[27] Bjorkbacka H, Kunjathoor VV, Moore KJ, Koehn S, Ordija CM, Lee MA (2004). Reduced atherosclerosis in MyD88-null mice links elevated serum cholesterol levels to activation of innate immunity signaling pathways. Nat Med..

[28] Rakoff-Nahoum S, Medzhitov R (2008). Innate immune recognition of the indigenous microbial flora. Mucosal Immunol..

[29] Rakoff-Nahoum S, Paglino J, Eslami-Varzaneh F, Edberg S, Medzhitov R (2004). Recognition of commensal microflora by Toll-like receptors is required for intestinal homeostasis. Cell..

[30] Ridker PM, Rifai N, Stampfer MJ, Hennekens CH (2000). Plasma concentration of interleukin-6 and the risk of future myocardial infarction among apparently healthy men. Circulation..

[31] Naya M, Tsukamoto T, Morita K, Katoh C, Furumoto T, Fuiji S (2007). Plasma interleukin-6 and tumor necrosis factor-alpha can predict coronary endothelial dysfunction in hypertensive patients. Hypertens Res..

[32] Esteve E, Castro A, Lopez-Bermejo A, Vendrell J, Ricart W, Fernandez-Real JM (2007). Serum interleukin-6 correlates with endothelial dysfunction in healthy men independently of insulin sensitivity. Diabetes Care..

[33] Hung MJ, Cherng WJ, Hung MY, Wu HT, Pang JH (2010). Interleukin-6 inhibits endothelial nitric oxide synthase activation and increases endothelial nitric oxide synthase binding to stabilized caveolin-1 in human vascular endothelial cells. Hypertension..

[34] Moss MB, Brunini TM (2004). Soares De Moura R, Novaes Malagris LE, Roberts NB, Ellory JC, et al. Diminished l-arginine bioavailability in hypertension. Clin Sci..

[35] Hadi HA, Carr CS, Al SJ (2005). Endothelial dysfunction: cardiovascular risk factors, therapy, and outcome. Vasc Health Risk Manag..

[36] Dickhout JG, Austin RC (2006). Proteasomal regulation of cardiac hypertrophy: is demolition necessary for building?. Circulation..

[37] Freigang S, Ampenberger F, Weiss A, Kanneganti TD, Iwakura Y, Hersberger M (2013). Fatty acid-induced mitochondrial uncoupling elicits inflammasome-independent IL-1alpha and sterile vascular inflammation in atherosclerosis. Nat Immunol..

[38] Pattison MJ, Mackenzie KF, Arthur JS (2012). Inhibition of JAKs in macrophages increases lipopolysaccharide-induced cytokine production by blocking IL-10-mediated feedback. J Immunology..

[39] Forsberg G, Hernell O, Hammarstrom S, Hammarstrom ML (2007). Concomitant increase of IL-10 and pro-inflammatory cytokines in intraepithelial lymphocyte subsets in celiac disease. Int Immunol..

[40] Han X, Kitamoto S, Wang H, Boisvert AW (2010). Interleukin-10 overexpression in macrophages suppresses atherosclerosis in hyperlipidemic mice. FASEB J..

[41] Pinderski Oslund LJ, Hedrick CC, Olvera T, Hagenbaugh A, Territo M, Berliner AJ (1999). Interleukin-10 blocks atherosclerotic events in vitro and in vivo. Arterioscl Thromb Vasc Biol..

[42] Khan F, Patterson D, Belch JJ, Hirata K, Lang CC (2008). Relationship between peripheral and coronary function using laser Doppler imaging and transthoracic echocardiography. Cin Sci..

[43] Khan F, Belch JJ, MacLeod M, Mires G (2005). Changes in endothelial function precede the clinical disease in women in whom preeclampsia develops. Hypertension..

